# Influence of menstrual cycle and oral contraception on taxonomic composition and gas production in the gut microbiome

**DOI:** 10.1099/jmm.0.001987

**Published:** 2025-03-28

**Authors:** Fernanda Terrazas, Scott T. Kelley, Taylor DeMasi, Kristine Giltvedt, Michelle Tsang, Kaelyn Nannini, Mark Kern, Shirin Hooshmand

**Affiliations:** 1 Department of Biology, San Diego State University, San Diego, USA; 2 School of Exercise and Nutritional Sciences, San Diego State University, San Diego, USA

**Keywords:** breath hydrogen, breath methane, human gut microbiome, oral contraceptives, sex hormones

## Abstract

**Introduction.** Oral contraceptives (OCs) are widely used for birth control and offer benefits such as menstrual cycle regulation and reduced menstrual pain. However, they have also been associated with an increased risk of cancer and reduced bone mass density.

**Gap Statement.** While the gut microbiome is known to interact with endocrine factors, the impact of hormonal OCs on its composition and function remains underexplored. Additionally, we explore the relationship of OC use and the microbiome to gas production, which can cause symptoms and be indicative of poor health.

**Aim.** This study investigates the effects of OCs on the diversity and composition of the gut microbiome and its association with breath hydrogen (H_2_) and methane (CH_4_) levels.

**Methodology.** We utilized 16S rRNA gene sequencing to analyse faecal samples from 65 women, comparing OC users with non-users at two menstrual cycle time points. Breath tests measured hydrogen and CH_4_ production. Data were analysed for microbial diversity, community composition and correlation with gas production.

**Results.** There were no differences in overall microbial diversity between OC users and non-users in samples collected on day 2 of the menstrual cycle. However, on day 21, we found a significant difference in microbial richness, suggesting a cycle-dependent effect of OCs on gut microbiota species richness but not composition. We found a strong correlation between H_2_ and CH_4_ concentrations and an interaction between OC use and the menstrual cycle on H_2_ and CH_4_ production. We also identified several taxa associated with both high levels of H_2_ and CH_4_ production and OC use.

**Conclusion.** Our study highlights the intricate relationships among hormonal contraceptives, the gut microbiota and gas production and connects shifts in the microbiome composition to gastrointestinal symptoms (e.g. gas production) that can impact overall health. This underscores the need for further research on the long-term effects of OCs and for the development of precise therapeutic strategies to address potential adverse effects. Our findings offer new perspectives on the microbiome–hormone–gas production nexus, potentially broadening our understanding of the systemic implications of OCs.

­

**Impact Statement**

The study advances the understanding of how oral contraceptives (OCs) affect the gut microbiome, an area with limited prior research. It delineates the impact of OCs on the diversity and composition of the gut microbiome, especially in relation to gas production. Our findings are significant for women of reproductive age using OCs, as they could link changes in the microbiome to potential gastrointestinal symptoms and alterations in microbial-induced gas profiles. Researchers and medical professionals can use findings as a basis for further studies on the interactions between hormones and the gut microbiome, which could be valuable for advising patients about potential side effects of long-term OC use. The study identifies specific microbial taxa that are associated with OC use, contributing new knowledge to the field of medical microbiology and reproductive health. It provides new evidence on the interplay between OCs and the gut microbiome, potentially influencing future guidelines on OC usage and informing targeted interventions to mitigate adverse gastrointestinal effects. The implications extend beyond contraception, touching on broader aspects of women’s health and hormonal management.

## Data Summary

The authors confirm all supporting data, code and protocols have been provided within the article or through supplementary data files.

## Introduction

Oral contraceptives (OCs) are one of the most commonly prescribed methods of birth control in the USA, with ~12.6% of women of reproductive age using them as their preferred method [1]. OC pills generally consist of natural or synthetic versions of the hormones oestrogen and progestin to prevent pregnancy through different mechanisms of action. These include the inhibition of ovulation, alteration of the endometrium and thickening of cervical mucus [2]. Beyond contraception, OCs offer additional benefits, including the regulation of menstrual cycles, reduced menstrual pain and acne and lower risk of issues like ovarian and endometrial cancer. Nonetheless, they carry potential drawbacks. Long-term use of OCs may increase the risk of breast, liver and cervical cancers [3] and potentially reduce bone mineral density [4,5].

The human gastrointestinal tract harbours a vast and dynamic assembly of micro-organisms, collectively referred to as the gut microbiome. This complex ecosystem, primarily composed of bacteria, serves as an integral determinant of health, as it can both substantially influence and be influenced by multiple physiological systems. Beyond its fundamental role in digestion and nutrient absorption, the gut microbiome plays a crucial role in immune response and facilitates communication within the gut–brain axis [6,7]. In addition, the microbiome may also influence endocrine activity. For instance, gut bacteria can promote the release of hormones from enteroendocrine cells via metabolites [8,9], metabolize hormone-like chemicals and even contribute to neurohormone production [10]. Moreover, bacteria can regulate sex hormones through the activity of specific enzymes [11], which, in response, can influence the composition and function of the microbiome [12].

Emerging research discussing the link between sex steroids and the human gut microbiome from several observational studies involving postmenopausal women or women using OCs was reviewed previously [13]. For instance, Flores *et al*. showed that species richness and diversity might impact systemic oestrogen levels, with pronounced associations in men and postmenopausal women [14]. Their results proposed that gut microbiota might primarily influence systemic oestrogen levels, an idea supported by Fuhrman *et al*. [15]. Changes in gut microbiota are associated with hyperandrogenism in humans and mouse models [16,18]. Rizk and Thackray suggested that elevated testosterone levels may play a role in regulating the composition of the gut microbiome in women [16]. Despite the growing recognition of the microbiome and hormone interaction, studies investigating the impact of hormonal OCs on the female microbiome are limited. Mihajlovic *et al*. found reduced gut microbiota richness in women using OCs, potentially due to reduced oestradiol and progesterone levels [19]. Furthermore, a longitudinal study identified an association between endogenous sex hormones and the microbiome’s composition and function following OC use [20].

In the process of fermentation, micro-organisms in the gut break down substances within the anaerobic environment of the digestive tract. This leads to the production of numerous volatile compounds as by-products or intermediates. Most of these compounds are gases such as hydrogen (H_2_), carbon dioxide (CO_2_) and methane (CH_4_). However, there are also trace amounts of gases like hydrogen sulphide (H_2_S), methanethiol (CH_3_SH) and dimethyl sulphide (C_2_H_6_S), along with short-chain fatty acids such as acetate, propionate and butyrate [21,22]. Different species and strains of bacteria produce varying amounts and types of gases, often reflecting their metabolic capabilities and dietary inputs [21,23]. While intestinal gas is normal for most healthy people, it has been associated with abdominal symptoms such as bloating, constipation, abdominal pain and excessive passing of gas. These symptoms, while occasionally reflective of physiological variations, can also signify chronic conditions such as irritable bowel syndrome or other functional gastrointestinal disorders [21,24]. Alterations in the composition of the gut microbiota – potentially induced by factors such as hormonal contraceptives – could exacerbate gastrointestinal symptoms or alter gas profiles, particularly in susceptible individuals or those with pre-existing conditions. Thus, a secondary goal of this project was to determine if shifts in the gut microbiota could be detected through changes in the excretion of key gut-derived gases, which can be measured via exhaled breath.

In this study, we leveraged 16S rRNA gene sequencing data to investigate the impact of OCs on the diversity and composition of the gut microbiome and to explore whether these changes are associated with H_2_ and CH_4_ production. To account for the dynamic nature of the microbiome across different phases of the menstrual cycle, we collected faecal and breath gas samples in OC users and non-OC users on day 2 and day 21 of the cycle. Our results revealed important interactions between groups and menstrual cycle day on gut microbial diversity and gas levels, highlighting potential shifts in specific microbial populations and gas production. Our findings could offer novel insights that could pave the way for targeted interventions to enhance gut health.

## Methods

This study was granted approval by the Institutional Review Board at San Diego State University, under IRB protocol #HS-2020–0070. A cohort of 65 female participants was recruited, and after each individual gave informed consent to take part in the research, they were enrolled in the study. Each of them was provided with screening forms enumerating requirements for OC use duration, health conditions, medications and exclusionary criteria. All participants used monophasic pills with different levels of oestrogen (specifically 20, 30 and 35 mcg), and the distribution of the various doses was similar among the OC users. Eligibility was determined by the following conditions: body mass index range from 18 to 32 and age between 18 and 25 years. Individuals with a medical history of cancer, metabolic bone disease, renal disease or those undergoing treatments involving anabolic agents, endocrine or neuroactive drugs were excluded from the study. All participants’ bone density was measured using dual-energy X-ray absorptiometry, and there were no differences among the study participants between control and OC users [25]. Additionally, those exceeding two alcoholic drinks per day and smokers were excluded. For inclusion in the OC user group, participants had to have been on OCs for a period ranging between 1 and 5 years. Conversely, non-OC users (control) were required to have refrained from OCs for at least 3 months prior to their involvement in the study. A total of 64 faecal samples were collected from eligible participants on day 2 and 31 samples on day 21 of their menstrual cycles and were stored at −80 °C until processing. In addition to the collection of faecal samples, a breath test was conducted to examine the effect of OCs on the production of H_2_ and CH_4_ via fermentation in the gut. Initially, two baseline samples were obtained, after which participants ingested lactulose (10 g), a non-digestible sugar solution, to enable monitoring of its transit through the small intestine. Two alveolar breath samples were collected every 15 min for 120 min and analysed using a calibrated QuinTron BreathTracker SC Analyzer system (Milwaukee, WI). The resultant H_2_ and CH_4_ readings were reported in p.p.m. and logged for subsequent analyses.

### DNA isolation

Briefly, ~250 mg of faecal sample was weighed and transferred to a sterilized microcentrifuge tube; the remaining sample was stored at −80 °C. Ethanol was removed from samples through centrifugation at 14,000 ***g*** for 2 min, followed by the disposal of the supernatant. This process was repeated two to three times, depending on sample consistency, to ensure most of the ethanol was removed. Residual ethanol was allowed to evaporate at room temperature for 10 to 15 min. Genomic bacterial DNA was isolated and purified from ethanol-dried samples using the QIAGEN DNeasy Powersoil Pro Kit (Catalogue 47016, QIAGEN LLC, Germantown, MD) following the manufacturer’s protocol. DNA was extracted from faecal samples, two positive extraction controls (25 µl of ZymoBIOMICS^™^ Microbial Community Standard, Cat. No. D6300) and two negative controls (250 µl of H_2_O). Extracted DNA samples were stored at −80 °C until further processing.

### PCR amplification and sequencing

The V4 region of the 16S rRNA gene was amplified using barcoded bacterial primers 515F (GTGCCAGCMGCCGCGGTAA) and 806R (GGACTACHVGGGTWTCTAAT) [26,28], which allow for pooling of multiple PCR amplicons in a single sequencing run. Extracted DNA samples, two positive PCR controls (ZymoBIOMICS^™^ Community DNA Standard, Cat. No. D6305) and two negative controls were included. Each PCR reaction consisted of 2.5 µl of 1 µM forward primer, 2.5 µl of 1 µM reverse primer, 6.5 µl of water and 1 µl of extracted DNA. PCR thermocycling conditions were as follows: initial denaturing at 94 °C for 3 min, 25 cycles of amplification at 94 °C for 45 s, 50 °C for 1 min and 72 °C for 90 s. Finally, samples were held at 72 °C for 10 s, followed by a hold at 4 °C. PCR amplicon sequence libraries were prepared at the Scripps Research Genomics Core Facility (San Diego, CA) and sequenced on the Illumina NextSeq 2000 platform. A total of 125 sequencing libraries were generated, comprising both controls and faecal samples. Gut microbial diversity profiles were generated from samples collected from 66 subjects and classified per group as ‘Control’ and ‘OC user’. The control group included 36 samples from women who did not use OCs. The OC user group, on the other hand, consisted of 84 samples from women who used OCs. Samples were also classified based on the day of collection as ‘day 2’ and ‘day 21’.

### Amplicon sequence processing and analysis

FASTQ files were imported to QIIME2 (v. 2022.8.0) and demultiplexed using the q2-tools-import and q2-demux-single scripts correspondingly. Sequences underwent initial quality filtering with q2-q-score prior to denoising with q2-denoise-16S from the Deblur plugin [29] to obtain amplicon sequence variants (ASVs). Based on quality scores, forward reads were truncated at position 230. Taxonomy assignment was determined using q2-classify-sklearn and the 515f-806r-human-stool-classifier trained on Silva 138.1 [30]. Taxonomic distributions of each sample were calculated using the q2-taxa-barplot script. The microbiota present in the faecal samples were representative of the human gut flora, as confirmed by the computed visualizations. While the taxonomic analysis indicated low levels of contamination based on positive control standards, the q2-filter-samples script was used to eliminate any features inconsistent with those found in the positive controls. CurvCut was used to remove features that were present in two or fewer samples to help mitigate overdispersion in subsequent analyses. CurvCut’s heuristic approach depends on the feature distribution of the data, so the recommended cutoff value differs by dataset [31].

The q2-diversity core-metrics-phylogenetic script was used to calculate alpha and beta diversity metrics, using a rarefaction depth of 1,473. Consequently, this resulted in excluding five samples that contained fewer than 1,473 sequences each. To assess the microbial community diversity within samples, four alpha diversity metrics were computed: Shannon entropy (SE), Observed features (OF), Faith’s diversity (FD) and Pielou’s evenness (PE). To compare beta diversity (diversity between samples), count data were processed through a zero-replacement procedure using a pseudo-count of 0.001, followed by a centered-log-ratio (CLR) transformation. A non-metric multidimensional scaling (NMDS) ordination was generated using Euclidean (CLR-transformed ASV abundances) distances. The ordination was visualized via NMDS plots created with ggplot2 in R (v. 3.4.2). Data points, which represent samples, were coloured by group.

### Statistical analysis

Statistical analyses were conducted using the RStudio statistical package (v. 2023.3.1.446). Normality of the data was confirmed with the Shapiro–Wilk test, and any non-normally distributed variables were transformed accordingly. Within-sample diversity comparisons were evaluated using linear mixed effects models (LMEMs) using the lme4 package in R (lme4 v. 1.1.33). The fitted model included group, day and their interaction as predictor variables, while subject was included as random effect to accommodate within-subject correlations in the data. For between-sample diversity comparisons, a permutational multivariate analysis of variance (PERMANOVA) was performed using 9,999 permutations (vegan package v. 2.6.4) with Euclidean distance measures. Finally, beta dispersion, which represents the distance from the centroid of each group, was analysed for each group. This was achieved by using the distances obtained from the PERMANOVA analysis, followed by a permutation-based test of multivariate homogeneity of group dispersions.

The machine learning approach coda4microbiome (v. 0.1.4) for compositional data was used to determine sets of species that, based on their relative abundances, collectively distinguish microbiome samples by group, as well as H_2_ and CH_4_ breath levels. The algorithm uses penalized logistic regression to compute microbial balances, coefficients and area under the curve (AUC) values, thereby identifying the simplest microbial balances that depict variances across different microbial communities [32]. The resulting output is a log-contrast model that presents balances between two distinct microbial communities. In this context, a positive microbial coefficient implies that a particular microbe contributes to the community with a higher balance. Conversely, a negative microbial coefficient suggests that the microbe contributes less, resulting in a community with a lower balance. The coefficient’s absolute value represents the relative contribution of the microbe to the balance model. AUC values for H_2_ and CH_4_ were computed to summarize gas production over time for each subject to identify said microbial balances. Additional differential abundance analyses were performed using ALDEx2 [33] and ANCOMBC [34] (v. 2.2.2 and v. 1.32.0, respectively). These methods allowed for identification of differentially abundant taxa while accounting for compositional and sampling biases inherent to microbiome data (see Figs S2–S4, accessible at 10.5281/zenodo.14722217, available in the online Supplementary Material). Moreover, to evaluate the impact of OCs on H_2_ and CH_4_ levels, an LMEM was computed for each gas. Each model accounted for group and day, as well as their interaction, as predictive factors, while incorporating subject as a random effect.

In addition, to uncover associations between microbiome composition and breath gas production, Spearman’s rank correlation coefficients were calculated. This analysis assessed not only the relationship between H_2_ and CH_4_ levels but also explored how microbial abundances correlate with each individual gas and their ratio (CH_4_:H_2_). This non-parametric alternative, chosen due to its reliability with non-linear data, enabled the identification of specific microbial taxa whose abundances are linked to variations in gas production.

## Results

### Sample filtering

Illumina sequencing yielded around 1.6 million sequences spanning all samples, with a mean of 13,185 features per sample and a range of 551,312 to 47 features. According to ZymoBIOMICS^™^, community standards are certified to contain<0.01 % of foreign contaminants, meaning that alien taxa present at>0.01 % can be attributed to contaminants introduced by the processing workflow. Based on community distribution plots, the positive controls used exhibited ~0.83% of contamination each. Features detected in negative controls and those inconsistent with the theoretical composition of the community standards were removed in accordance with the defined threshold. Some of these features belonged to the *Bacteroides*, *Blautia* and *Prevotella* genera. These sequences were removed one by one while inspecting the taxa distribution per sample before and after filtering to ensure that their removal would not affect the overall remaining distribution. While this decision led to the removal of sequences commonly found in the gut, it was consistent with methodological approaches taken to address potential contamination [35]. Following the application of CurvCut [31], features found in two or fewer samples were removed. Additionally, five samples with low sequence coverage (<1,473) were excluded: two from the ‘Control’ group on day 21 and two from the ‘OC User’ group on day 21.

### Alpha diversity

**Fig. 1.**
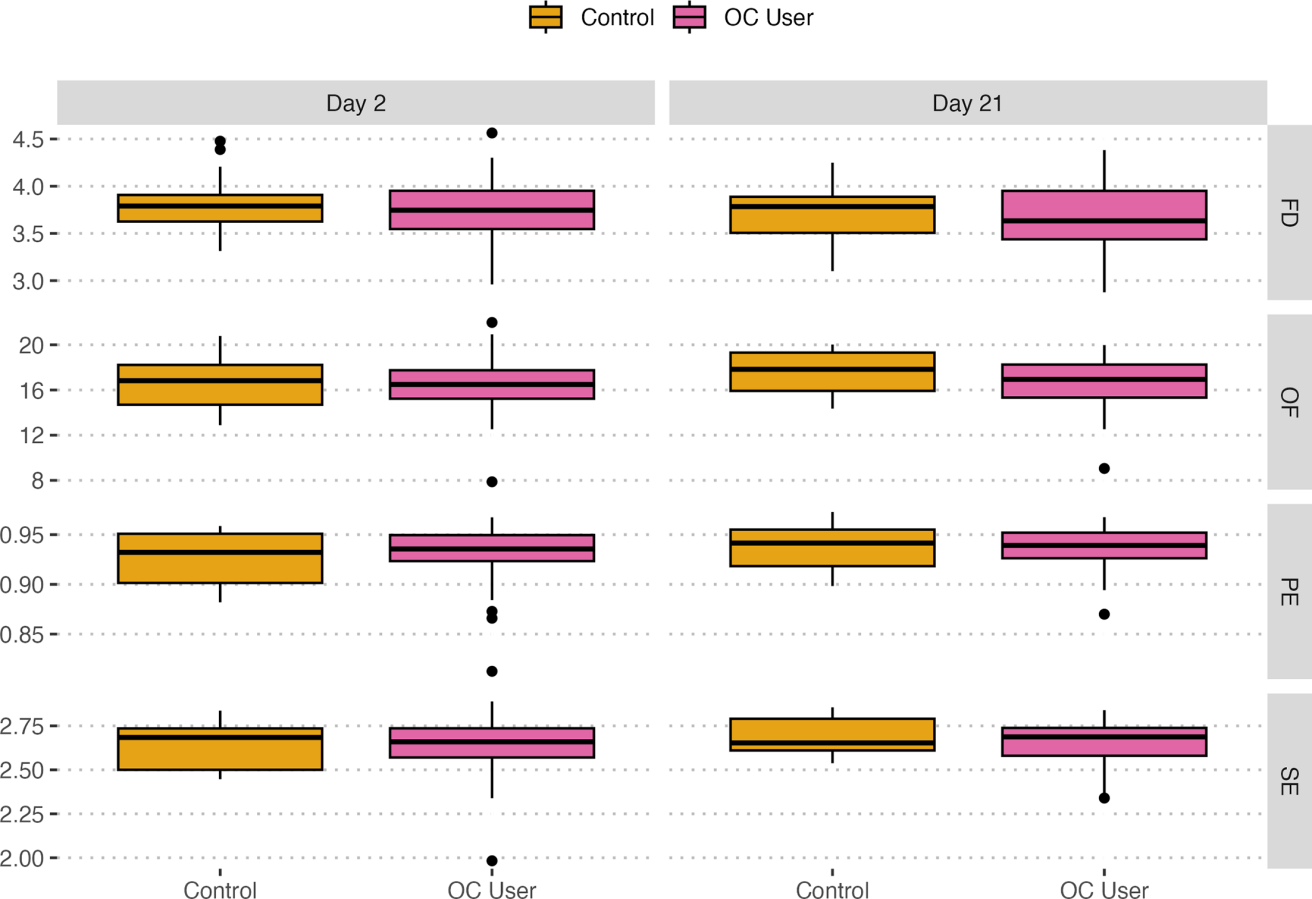
Distributions of four distinct alpha diversity indices – Faith’s diversity (FD), Observed features (OF), Pielou’s evenness (PE) and Shannon entropy (SE) – were evaluated on day 2 and day 21 for OC users (pink) and control group (yellow). No statistically significant differences were observed in the alpha diversity metrics between the groups, except for OF, which showed a significant interaction between group and day (*P*=0.04) according to LMEM results in Table 1.

Alpha diversity was evaluated using SE, OF, FD and PE. According to Fig. 1, gut microbial diversity was similar between groups. Accordingly, LMEMs revealed no statistically significant differences between groups among all alpha diversity metrics used except for OF, where the model found a significant interaction between group and day (*P*=0.04), suggesting that the OC effect on OF was more pronounced on day 21 (Table 1). However, fixed effects alone seem to explain only a small portion of the variance compared to random effects overall.

**Table 1.** Alpha diversity results from LMEM analyses

			*R* ^2^	
**Model**	**Chi-Sq**	***P*-value**	**Marginal**	**Conditional**
Shannon~GroupDayGroup: Day	0.4430.6441.445	0.5060.4220.229	0.016	0.477
OF~GroupDayGroup: Day	1.5460.0694.235	0.2140.793**0.040**	0.0348	0.561
FD~GroupDayGroup: Day	1.6161.5950.043	0.2040.2070.837	0.029	0.088
Piealou’s~GroupDayGroup: Day	0.0251.9160.065	0.8750.1660.799	0.014	0.356

### Beta diversity

**Fig. 2.**
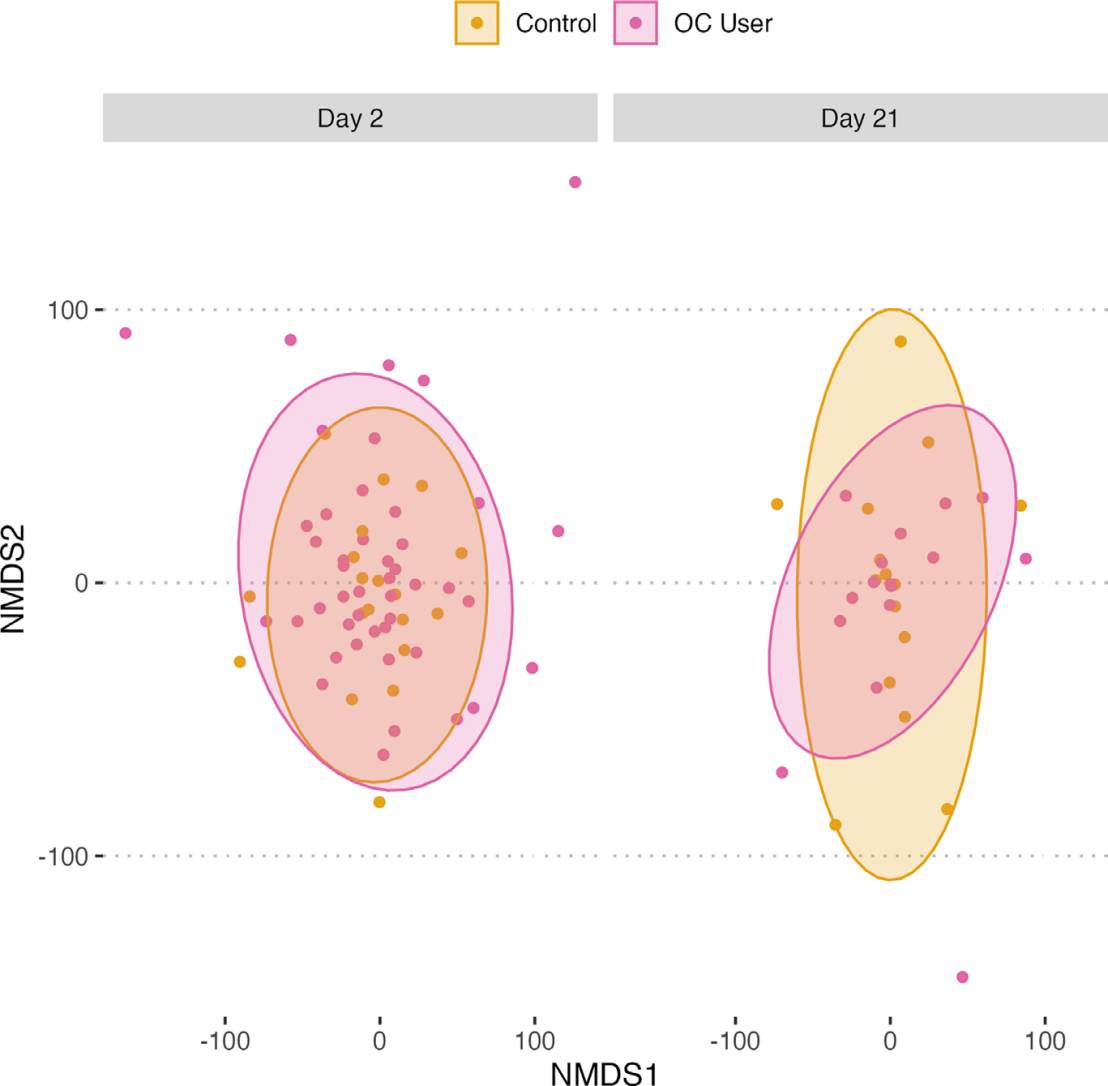
NMDS ordination depicting beta diversity of gut microbiome from OC users (pink) and control group (yellow) shows a scattered distribution without clear separation between the OC user and control groups, suggesting a lack of distinct microbial community structures. This was corroborated by a PERMANOVA test, which did not detect statistically significant differences in beta diversity between the two groups (Table 2).

To assess gut microbial diversity between samples, Euclidean distances were computed to construct an NMDS ordination. This enabled the examination of potential distinctions between groups. To statistically test the differences within these categories, a PERMANOVA test was used. As depicted in Fig. 2, the NMDS ordination did not reveal any evident clustering of samples based on group. This observation was further substantiated by the PERMANOVA test, which found no statistically significant differences in community composition between said groups (Table 2).

**Table 2.** Beta diversity results from PERMANOVA analyses

Stress	Model	Sum of squares	*F*	*R* ^2^	*P*-value
0.274	ASVs~GroupDayGroup: Day	25,41626,45223,722	0.9631.0020.899	0.01030.01070.0096	0.6920.4470.948

### Differentiation of microbial abundances by group and gas levels

**Fig. 3.**
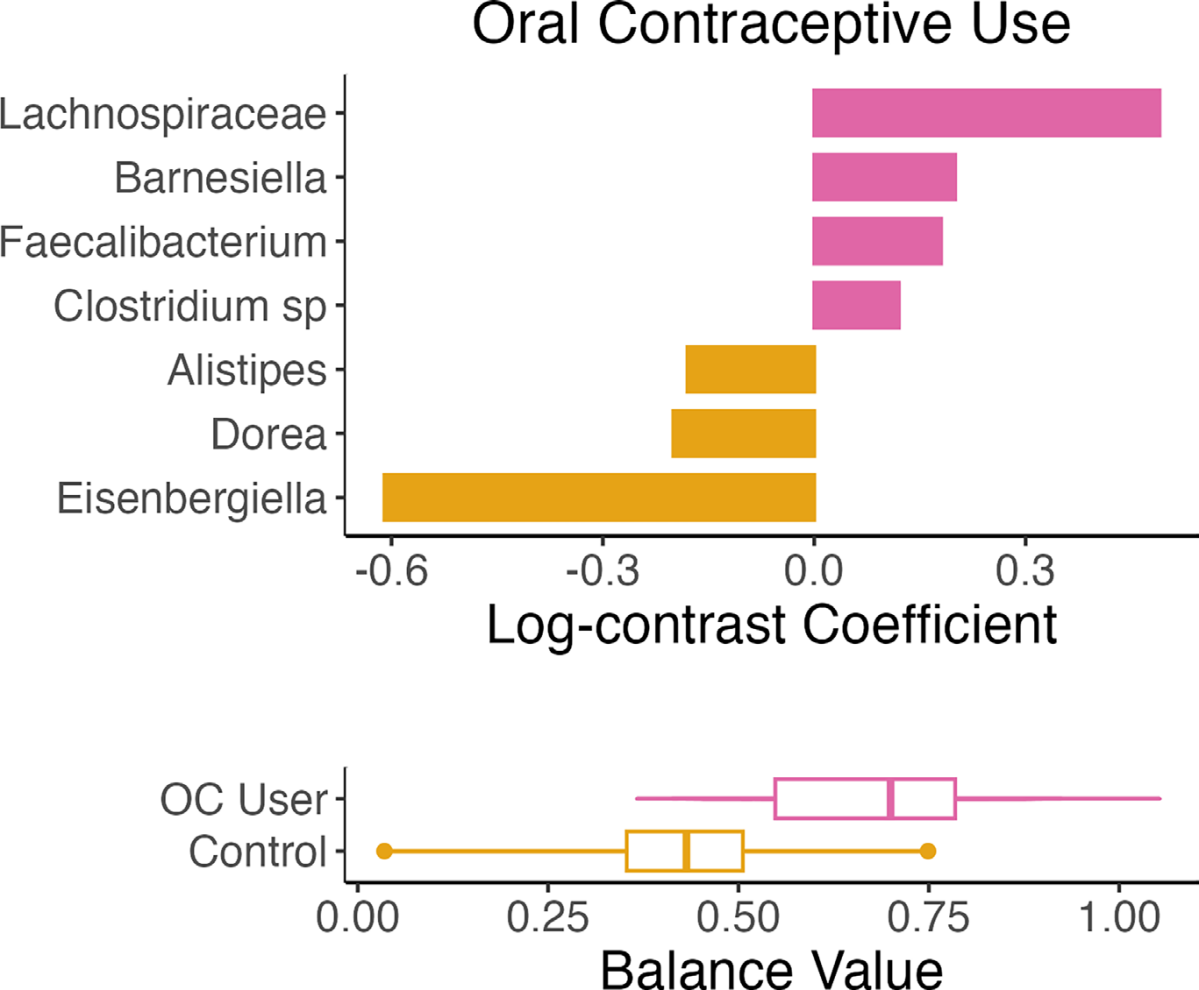
Microbial balances define differences between OC users and controls. The bar plot (top) illustrates the log-contrast coefficients for taxa derived from a penalized logistic regression model using the coda4microbiome algorithm. Taxa with coefficients signifying an absolute balance of at least 0.05 are shown, with the length of the bar representing the proportion of each taxon’s contribution. Taxa are ranked by their contribution to the balances, with positive coefficients indicating greater abundance and negative coefficients indicating lesser abundance in OC users. The box plot (bottom) shows the sample distribution of balance values for each specified group.

The coda4microbiome algorithm was used to identify microbial balances between OC user and control groups, as well as between high and low gas levels. Figs3^5^ illustrate the taxa that contributed most strongly to each balance. The orientation of the bar signifies the nature of the contribution (positive or negative) to the balance by each taxon and shows in which group the taxon was more abundant. We also applied two other compositionally aware methods, ANCOMBC and ALDEx2, to detect differential microbial abundances. ALDEx2 did not detect any significant differences, partly due to the number of abundance comparisons. The ANCOMBC results are presented in Figs S1–S4 (Relative abundances by sample are presented for control and OC users are shown in Fig. S5). For the purpose of this discussion, we focus on the coda4microbiome results.

**Fig. 4.**
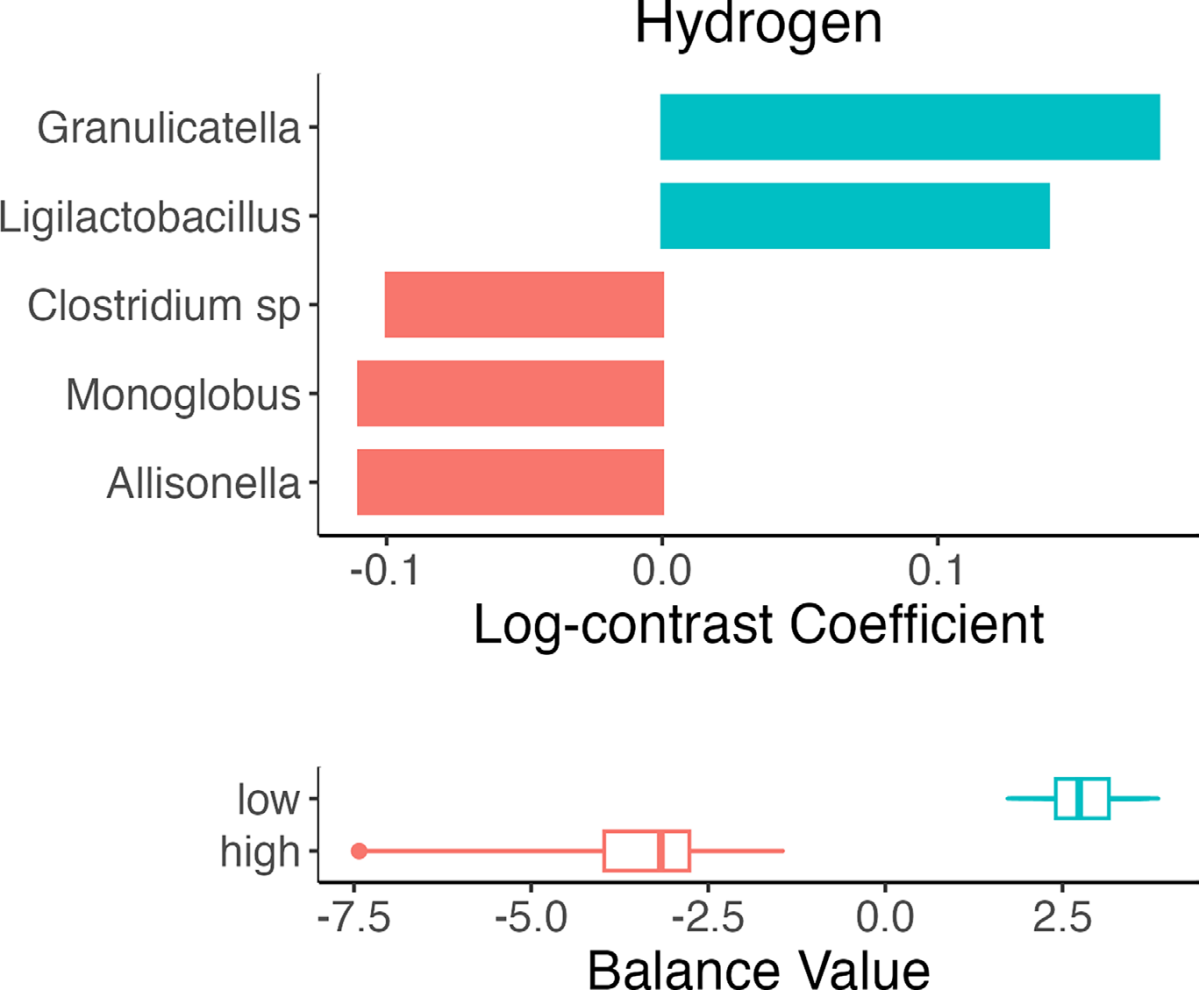
Microbial balances define differences between high and low H_2_ groups.

**Fig. 5.**
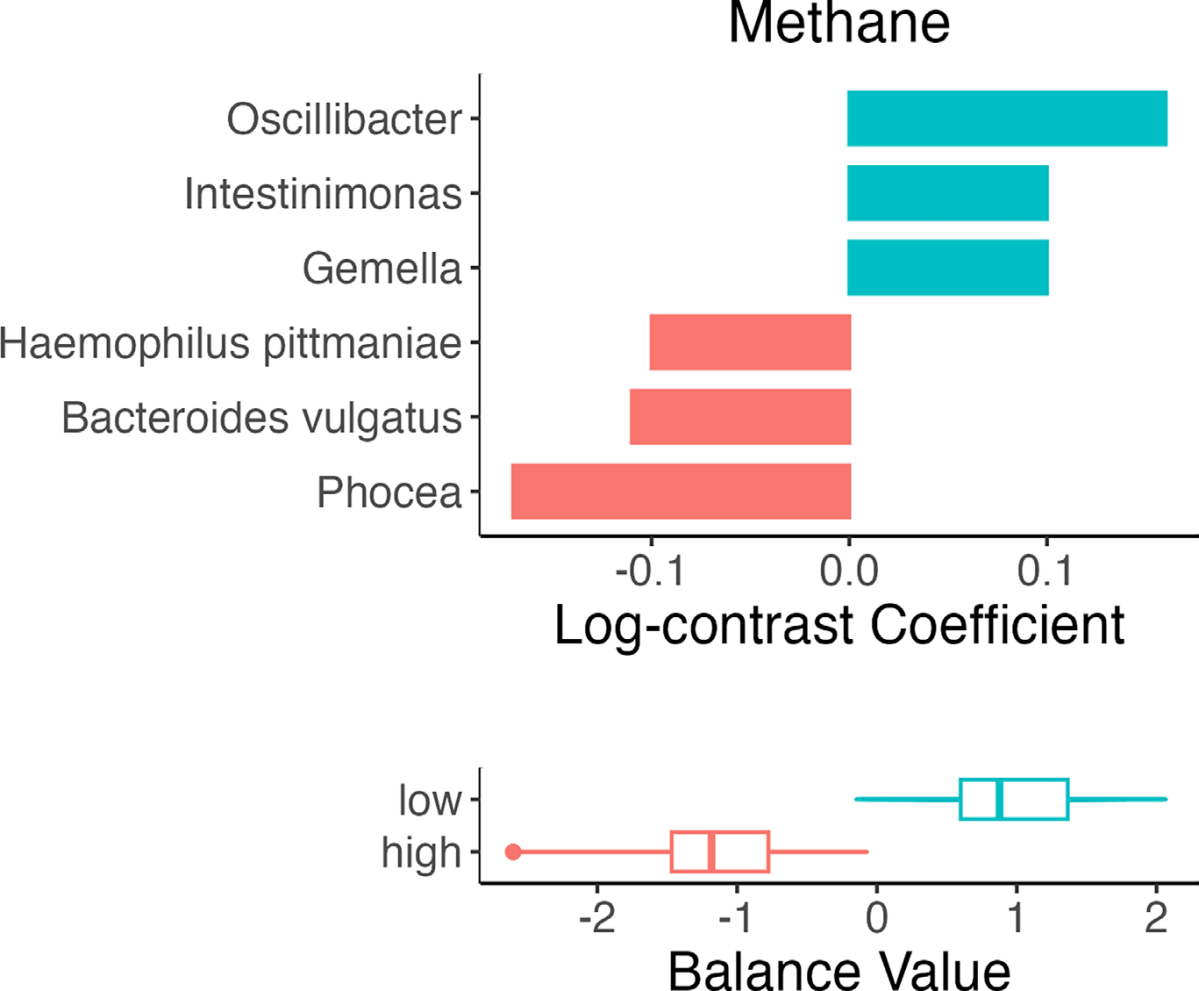
Microbial balances define differences between high and low CH_4_ groups.

In the control group, *Eisenbergiella* bacterium, followed by uncultured species of *Dorea* and *Alistipes*, contributed positively to the balance, which indicates that these taxa have a higher relative abundance in the control group when compared to the OC user group. In contrast, the OC user group had higher relative abundances of *Lachnospiraceae*, which had the highest contribution to the negative balance, followed by *Barnesiella*, *Faecalibacterium* and *Clostridium* sp. (Fig. 3).

Additionally, samples were classified based on whether they had high or low levels of H_2_ or CH_4_ and differentiated in the same manner. The ‘high’ H_2_ group suggested higher relative abundances of *Allisonella*, uncultured *Clostridiales* and *Clostridium*, while the ‘low’ H_2_ group was differentiated by *Granulicatella* and *Ligilactobacillus* (Fig. 4). The ‘high’ CH_4_ group was differentiated from the ‘low’ CH_4_ group for having greater relative abundances of *Oscillobacter*, *Intestimonas* and *Gemella*, while the ‘low’ CH_4_ had higher relative abundances of *Phocea*, *Bacteroides vulgatus* and *Haemophilus pittmaniae* (Fig. 5).

### Group effect on H_2_ and CH_4_ levels

**Fig. 6.**
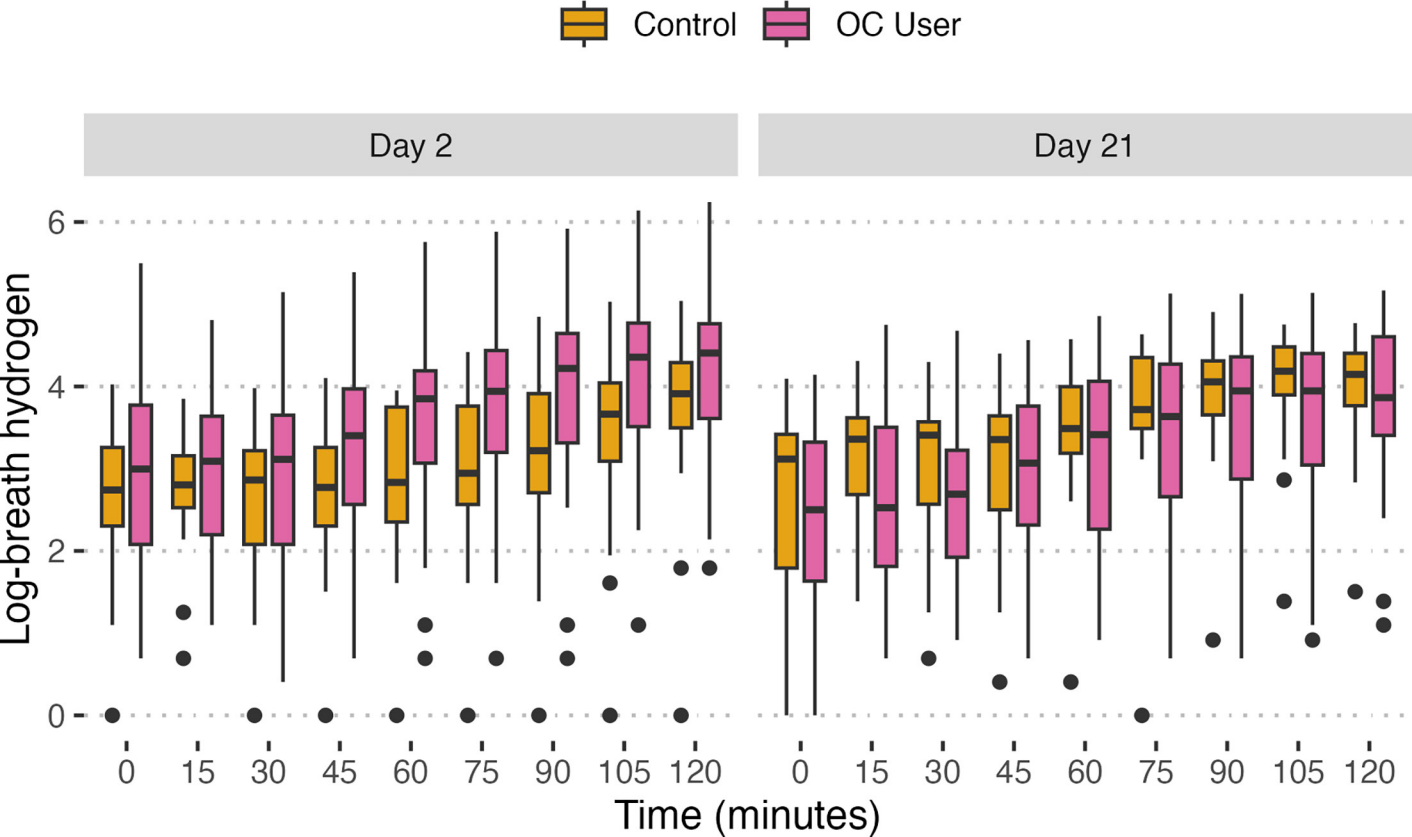
Comparison of breath H_2_ (ppm) levels in OC users (pink) and control group (yellow) on day 2 and day 21. Box plots display log-transformed breath H_2_ concentrations at each time point (in minutes) over a 2 h period following the ingestion of lactulose. Our LMEM revealed a statistically significant interaction between OC users and day on H_2_ levels (*P*<0.001), indicating a distinct response to lactulose at different cycle stages that is not present when considering group or day alone.

LMEMs were used to assess the effect of OCs on H_2_ and CH_4_ levels shown in Figs6  7. A model for each predicted variable (H_2_ and CH_4_) was fitted with group, day and their interaction. Random intercepts were incorporated for subjects to account for individual variability and within-subject correlation that arise from repeated measures (Table 3). The analysis revealed a statistically significant interaction between group and day on H_2_ levels (*P*<0.001), but when looking at group and day alone, neither displayed a significant effect on their own. For CH_4_ levels, the group alone did not significantly affect CH_4_ concentrations; however, the effect of day and its interaction with the group were statistically significant (*P*<0.001 for both), suggesting a detectable change in CH_4_ levels across days 2 and 21. Notably, the random effect explained a larger proportion of the variance compared to fixed effects in both scenarios.

**Fig. 7.**
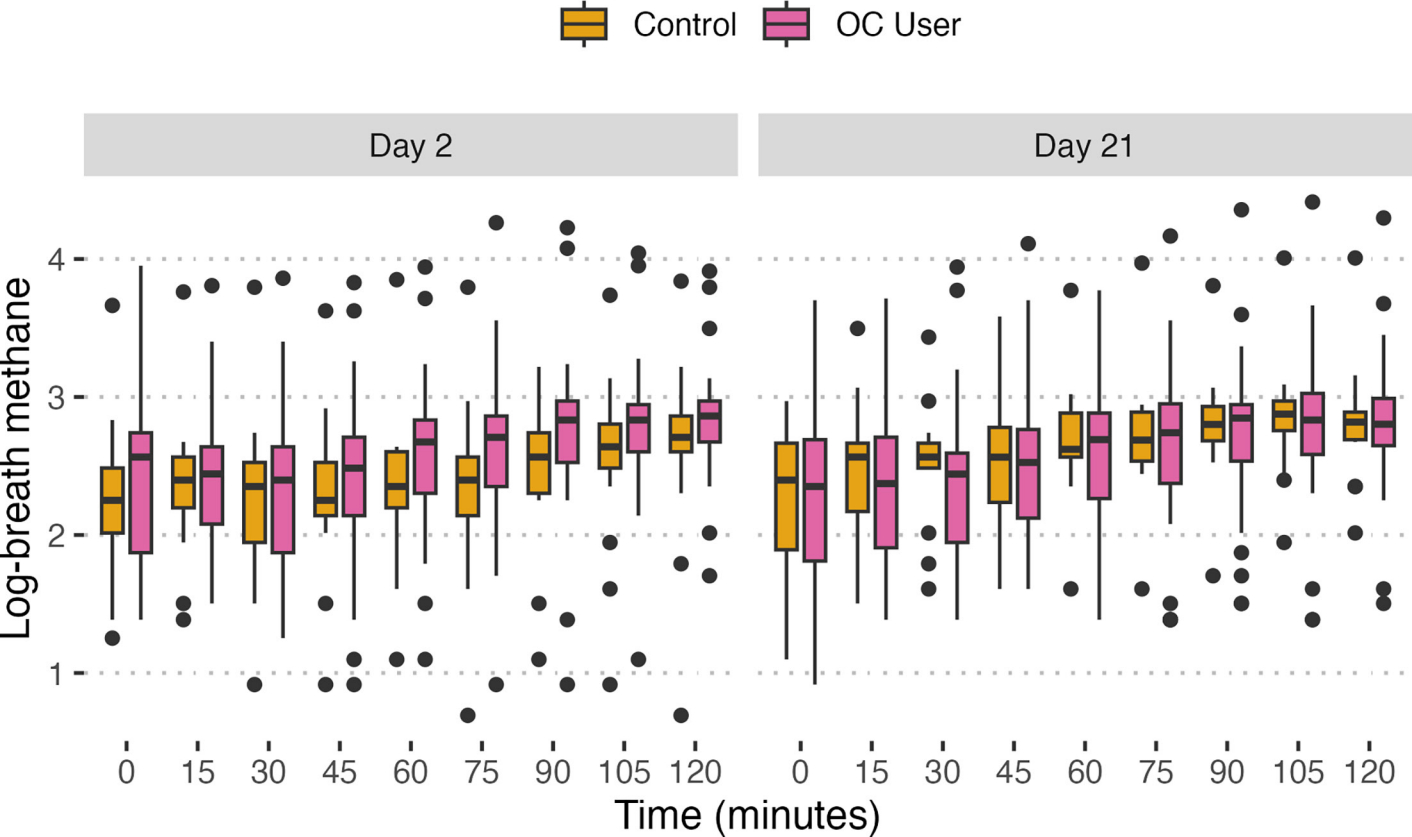
Comparison of breath CH_4_ (ppm) levels in OC users (pink) and control group (yellow) on day 2 and day 21. Box plots display log-transformed breath CH_4_ concentrations at each time point (in minutes) over a 2 h period following the ingestion of lactulose, indicating potential differences in CH_4_ production between the two groups at different phases of the menstrual cycle. According to our LMEM, both the effect of day and the interaction between group and day were significant (*P*<0.001).

**Table 3.** LMEM analysis of H_2_ and CH_4_ concentration measured during breath tests

			*R* ^2^	
**Model**	**Chi-Sq**	***P*-value**	**Marginal**	**Conditional**
H_2_~GroupDayGroup: Day	0.2540.32145.87	0.6140.571**<0.001**	0.038	0.426
CH_4_~GroupDayGroup: Day	0.10223.7626.00	0.271**<0.001<0.001**	0.018	0.614

### Hydrogen and methane correlations

**Fig. 8.**
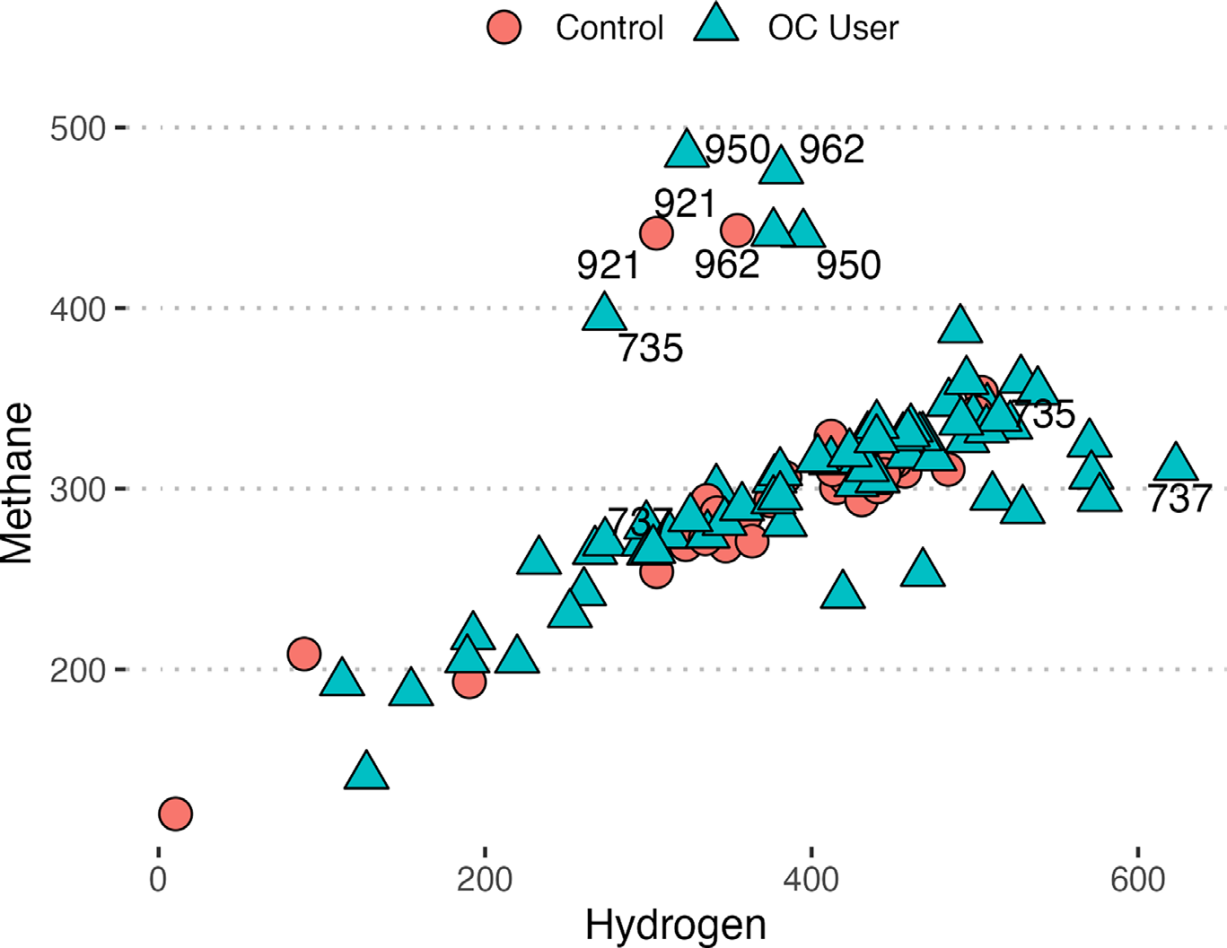
Positive correlation (*ρ*=0.724, *P*<0.001) between CH_4_ (ppm) and H_2_ (ppm) levels measured during breath tests across all subjects. Outliers are labelled based on subject number and, when excluded, the correlation coefficient increased to *ρ*=0.884.

The relationship between H_2_ and CH_4_ breath concentration levels was evaluated by calculating Spearman’s rank correlation coefficient. As shown in Fig. 8, H_2_ and CH_4_ measurements display a positive correlation with respect to one another (*ρ*=0.724, *P*<0.001).

Correlation analyses were also conducted to explore the association between microbial abundances and H_2_ and CH_4_ breath concentration, as well as the CH_4_:H_2_ ratio. These analyses identified several statistically significant correlations, both positive and negative, which are summarized in Tables4^6^. Additionally, some taxa were identified across the different analyses. Notably, uncultured *Dorea* was found to be positively correlated with H_2_ and CH_4_ breath concentrations while being negatively correlated with the CH_4_:H_2_ ratio.

**Table 4.** Correlation between microbial abundances and breath H_2_ concentration *P*-values were not adjusted for multiple comparisons.

Taxa	Spearman’s *ρ*	***P*-value**
Uncultured *Dorea*	0.2987	0.0063
*Streptococcus oralis*	0.2357	0.0322
*Turicibacter*	0.2244	0.0416
*Butyricicoccus*	0.2218	0.0441
*Ruminococcaceae*	0.2191	0.0468
*Eubacterium coprostanoligenes* group	−0.2162	0.0498
*Ruminococcaceae*	−0.2214	0.0444
*Holdemania*	−0.2225	0.0434
Uncultured *rumen*	−0.2259	0.0402
*Defluviitaleaceae* UCG011	−0.2291	0.0374
*Butyricimonas*	−0.2365	0.0316
UCG-005	−0.2378	0.0306
*Butyricimonas*	−0.2453	0.0257
*Lachnospiraceae* UCG010	−0.2477	0.0242
*Eubacterium siraeum*	−0.2542	0.0207
*Roseburia hominis*	−0.2597	0.0180
*Paraprevotella clara*	−0.2705	0.0136
Uncultured *prokaryote*	−0.2886	0.0083
*Massiliprevotella massiliensis*	−0.2952	0.0069

**Table 5.** Correlation between microbial abundances and breath CH_4_ concentration The table presents correlations with the strongest positive and negative associations (highest positive and lowest negative rho values). *P*-values were not adjusted for multiple comparisons. A comprehensive list of all significant correlations identified between microbial abundances and breath CH_4_ concentration is available in Table SA, accessible at 10.5281/zenodo.14722217.

Taxa	Spearman’s *ρ*	***P*-value**
*Barnesiella*	0.3544	0.0011
*Alistipes shahii*	0.3079	0.0048
*Erysipelotrichaceae* UCG-003	0.2295	0.0371
*Oscillospiraceae*	0.2251	0.0410
*Subdoligranulum*	0.2167	0.0492
Uncultured *Dorea*	0.2164	0.0496
Uncultured *Clostridiales*	−0.4396	0.0000
*Roseburia hominis*	−0.4293	0.0001
*Anaerotignum lactatifermentans*	−0.4033	0.0002
*Rhodospirillales*	−0.3980	0.0002
*Dysgonomonas capnocytophagoides*	−0.3979	0.0002
*Faecalitalea*	−0.3958	0.0002
UCG-005	−0.3919	0.0003
*Gastranaerophilales*	−0.3878	0.0003
*Clostridium spiroforme*	−0.3806	0.0004
*Lachnospiraceae* UCG010	−0.3768	0.0005
*Rhodospirillales*	−0.3765	0.0005
DTU089	−0.3739	0.0005
Candidatus *Gastranaerophilales*	−0.3695	0.0006
Bacterium ic1391	−0.3694	0.0006

**Table 6.** Correlation between microbial abundances and CH_4_:H_2_ ratio *P*-values were not adjusted for multiple comparisons.

Taxa	Spearman’s *ρ*	***P*-value**
*Eubacterium siraeum*	0.3127	0.0041
*Butyricimonas*	0.3072	0.0049
*Massiliprevotella massiliensis*	0.2910	0.0078
Uncultured *prokaryote*	0.2694	0.0140
*Ruminococcaceae*	0.2538	0.0208
*Methanobrevibacter smithii*	0.2514	0.0221
*Eubacterium coprostanoligenes* group	0.2334	0.0340
Uncultured *rumen*	0.2252	0.0409
*Bacillus*	−0.2209	0.0450
*Haemophilus*	−0.2384	0.0302
*Intestinibacter*	−0.2509	0.0224
*Butyricicoccus*	−0.2784	0.0110
*Streptococcus oralis*	−0.2792	0.0108
*Ruminococcaceae*	−0.2893	0.0082
Uncultured *Dorea*	−0.2896	0.0081

**Fig. 9.**
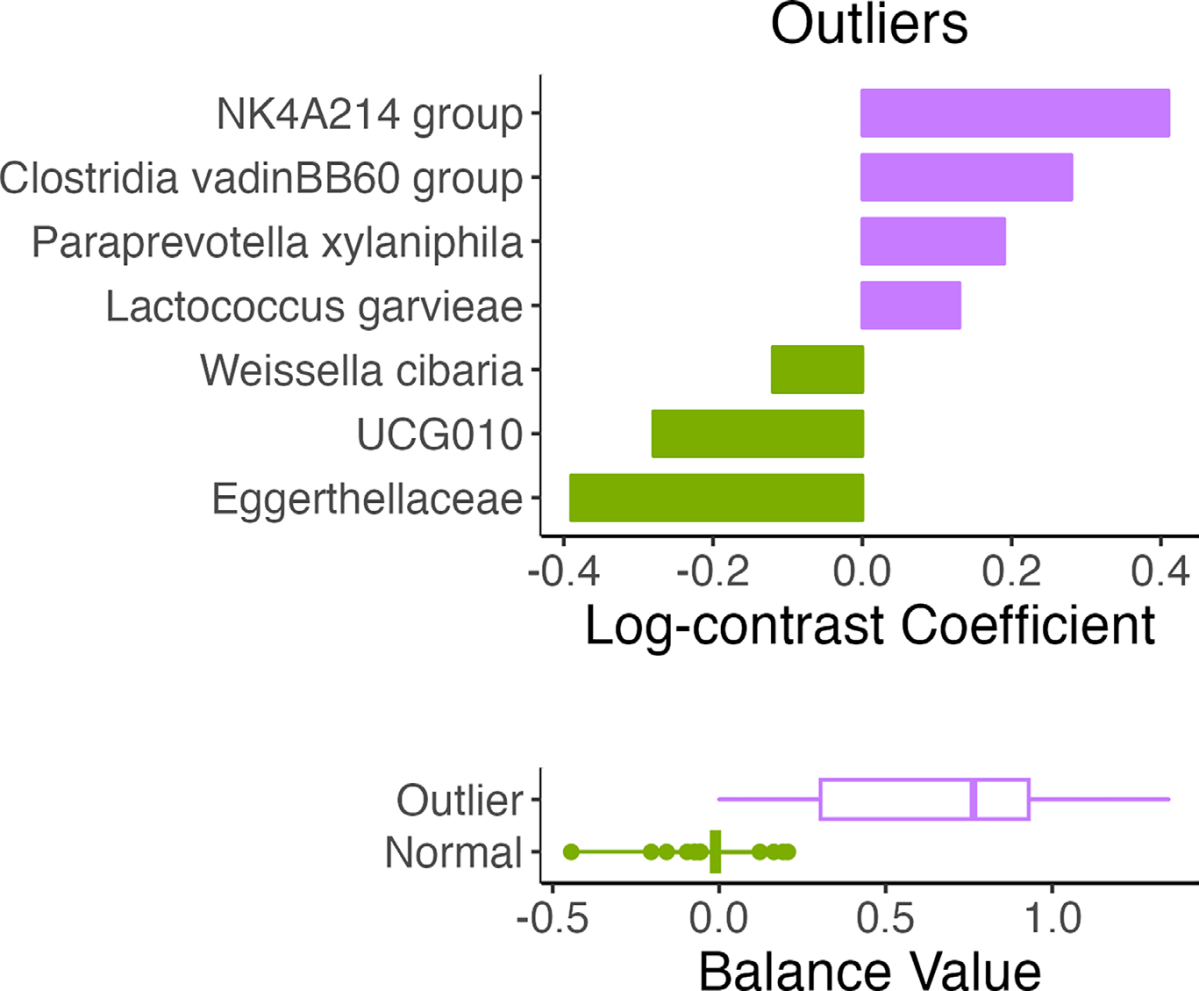
Microbial balances define differences between outlier and normal groups.

Samples that displayed extreme H_2_ and CH_4_ levels were grouped as ‘outliers’ and compared to the remaining samples (‘normal’) using the coda4microbiome algorithm (Fig. 9). NK4A214 group, *Clostridia vadinBB60* group, *Paraprevotella xylaniphila* and *Lactococcus garvieae* appeared to be present in higher relative abundances in the outlier group when compared to the normal group, which had higher relative abundances of *Eggerthellaceae*, *UCG010* and *Weissella cibaria* relative to the outlier group.

## Discussion

Our comparative analysis of bacterial diversity between women who were and were not taking OCs did not detect differences at the overall community level, for either between-sample (beta) or within-sample (alpha) diversity, except for OF (Table 1), where a model found a significant interaction between group and day (*P*=0.04). Our alpha and beta diversity results contrasted with two previous studies where differences in composition and diversity were strongly associated with OC intake [19] or with endogenous oestrogens [14]. However, a longitudinal study examining the effect of OC use on the gut microbiome also reported no detectable differences in beta diversity between OC users and the control group but found OCs to be associated with decreased alpha diversity over time [20]. Our samples were collected at the beginning of each woman’s menstrual cycle and then again on day 21. The interaction between group and day had a statistically significant effect on richness, where fixed effects coefficients suggested that OC use on ‘day 21’ was associated with a decrease in richness. This could be an indication of the fluctuations in hormone levels that occur during the menstrual cycle and that have also been shown to be affected by OC intake [19].

In contrast to the community-level results, our analysis revealed differences in the relative abundances of specific bacterial taxa between OC users and the control group. Notably, taxa such as *Lachnospiraceae*, *Barnesiella*, *Faecalibacterium* and *Clostridium* sp. showed higher relative abundances in the OC user group. Among these, *Lachnospiraceae* has been documented for its associations with female health and hormonal fluctuations. For instance, elevated levels of *Lachnospira* have been observed in postmenopausal women, suggesting hormone-related modulation that could also be influenced by OC use [36]. This sensitivity to hormonal changes is further evidenced by the altered abundance of this family in letrozole-treated mice [37]. Similarly, the increased presence of *Barnesiella* in the OC user group supports a study on pre-menopausal women using combined hormonal contraceptives that reported a higher abundance of this genus in the group using OCs [19]. Interestingly, *Lachnospiraceae* and *Barnesiella* were significantly associated with gas levels. *Lachnospiraceae* UCG010 was negatively correlated with H_2_ and CH_4_, while *Barnesiella* was positively correlated with CH_4_. Moreover, the link between *Faecalibacterium* and hormonal imbalance conditions, such as polycystic ovary syndrome (PCOS), could highlight its potential relevance in our findings, with prior research identifying *Faecalibacterium prausnitzii* as a distinguishing taxon in women with PCOS [17]. The association of *Clostridium* sp. with systemic oestrogen levels, demonstrated by its correlation with urinary oestrogens and faecal microbiome richness in men and postmenopausal women [14], suggests that OC-induced hormonal changes may influence the abundance of this genus as well, as highlighted in our OC group. These findings underline a distinctive impact of hormonal contraceptives on the gut microbiota, potentially mediated by the hormonal fluctuations induced by OC use.

Furthermore, our analysis comparing ‘high’ versus ‘low’ H_2_ groups also revealed differences in the microbial composition of the two. Specifically, the ‘high’ H_2_ group was characterized by a higher abundance of *Allisonella*, uncultured *Clostridiales* and *Clostridium* sp. While there is no existing evidence of a direct link between *Allisonella* and H_2_ production, its presence in co-culture with hydrogen-producing bacteria hints at its potential interaction within the microbial community in facilitating an environment conducive to the production of said gas [38]. Additionally, this taxon has been identified as a predictive marker in patients with type 2 diabetes (T2D) compared to normoglycaemic individuals and was also significantly associated with a higher score of obesity-related inflammation, suggesting its role in metabolic and inflammatory states [39,41]. In a 2013 study, non-alcoholic steatohepatitis patients had higher faecal abundance of *Allisonella* [42]. Moreover, *Clostridium* sp., found in our OC group and ‘high’ H_2_ group, including *Clostridia vadinBB60* group in our ‘outlier’ group, has been documented as a key player in the metabolic processes associated with H_2_ levels [43]. *Clostridia* have been identified as one of the main butyrate-producing classes, a process known to release free H_2_ [22,44]; additionally, *Clostridium spiroforme* was negatively associated with CH_4_ in our results. However, the dual nature of *Clostridium* sp., harbouring both pathogenic species and species beneficial to health, adds complexity to understanding its precise role in the microbiome [45]. Within our ‘outlier’ group, *P. xylaniphila*, a known succinate producer [46], was also present. The production of succinate, an intermediate in microbial propionate synthesis, also results in the release of H_2_ as a by-product [21]. This aligns with our findings on taxa associated with increased gas production, which underlines the intricacy of microbial metabolic pathways and their collective impact on H_2_ and, consequently, CH_4_ production.

In examining the composition of the microbiome in relation to CH_4_ production, our study delineates a distinction within the ‘high’ CH_4_ group, particularly highlighting an increased abundance of *Phocea*, *B. vulgatus* and *H. pittmaniae* relative to the ‘low’ CH_4_ group. Notably, the *Ruminococcaceae* family, to which the genus *Phocea* belongs, was significantly associated with H_2_ and the CH_4_:H_2_ ratio in our correlation analysis. This family is renowned for its butyrate-producing capabilities, which lead to the release of H_2_, a substrate for methanogenesis [22,47]. The association between high CH_4_ levels and a microbial consortium proficient in dietary fibre degradation, such as *Ruminococcaceae*, could suggest a synergistic relationship in the gut microbiota that favours CH_4_ production [48]. Interestingly, the genus *Phocea*, while not directly linked to H_2_ or CH_4_ production in available literature, has been implicated in studies exploring metabolic perturbations in conditions such as T2D and frailty, indicating its potential role in broader metabolic processes beyond CH_4_ production [49,50]. The significant presence of *B. vulgatus* in the ‘high’ CH_4_ group aligns with the understanding that *B. vulgatus* favours CH_4_ production, as it is a notable H_2_ producer [51]. Additionally, the increased abundance of *B. vulgatus* has been observed in women with PCOS, hinting at its involvement in hormonal and metabolic dysregulation [52]. *H. pittmaniae* has not previously been associated with CH_4_ production but has been documented to mediate both H_2_ uptake and production [53]. Our correlation analysis also found this species to be negatively associated with the ratio of CH_4_:H_2_. However, while we found no previous association of this genus with hormonal changes, a study focusing on the oral–gut axis in inflammatory bowel disease patients did find it to be associated with a ‘healthy gut state’ [54].

While *Methanobrevibacter smithii* – an archaeon known for its role in CH_4_ production from H_2_ [55] – was not significantly enriched in our ‘high’ CH_4_ or H_2_ groups, it was positively correlated with the CH_4_:H_2_ ratio (Table 5). Although our study focused on bacterial communities and our 16S primers were not specifically designed to amplify archaea, which makes this taxon unexpected, archaea in our samples may be of potential significance. The archaea are very understudied in the gut in general and could be playing a much larger role in dysbiosis than we collectively understand. Recent developments in the understanding of the human archaeome have underlined the importance of looking beyond bacteria and considering the role of methanogenic archaea. These suggest that archaea might be of higher relevance in methane-related conditions and in understanding what constitutes a healthy microbiome [56].

A study by Thomas *et al.* detected archaea in the gut of animals across the animal kingdom, vertebrates and invertebrates, and all the dominant gut lineages were associated with methanogenesis [57]. Another study showed a parallel phylogenetic relationship between methanogenic archaea and mammals dating back to their last common ancestor, further supporting the notion that archaeal methanogenesis is a core physiological process of the mammalian gut ecosystem [58]. This underscores the need to incorporate a direct investigation of gut archaea to better capture the complete microbial dynamics in the gut. Despite the ubiquity of archaea, they appear surprisingly rarely in gut metagenomic analyses, likely because of their low abundance relative to bacteria. Future studies on this topic will focus on investigating the diversity and abundance of gut methanogens in these samples using broad-spectrum archaeal 16S primers [57].

Finally, H_2_ and CH_4_ breath measurements were found to have a positive relationship. This result is inconsistent with studies that have found an inverse relationship between H_2_ and CH_4_, suggesting a symbiotic interaction between fermenting bacteria and hydrogen-consuming methanogens [59,60]. Anaerobic fermentation of undigested carbohydrates in the intestine generates H_2_, which serves as the material for CH_4_ production [55,61]. However, another study looking to link the gut microbiome with human breath CH_4_ and dietary fibre digestion also found positive correlations between H_2_ and CH_4_ [62]. Our LMEM analysis found that the interaction between group and day had a significant effect on both H_2_ and CH_4_ levels (*P*<0.001, respectively), indicating that the response to group may vary at different points in the menstrual cycle. While the day did not significantly influence H_2_ levels, it had a pronounced effect on CH_4_ production (*P*<0.001), possibly highlighting distinct temporal patterns in microbial metabolism of H_2_ and CH_4_. The overall results may reflect temporal variations in microbial activity or community composition between days 2 and 21 of the menstrual cycle. Interestingly, a 2020 study found that bowel habits and associated discomfort differ across menstrual phases, particularly noting increased discomfort on the first day of menstruation in healthy women taking OCs [63]. While this may extend to the observed temporal variation in our results, it should be noted that factors unique to each subject had considerable impact on H_2_ and CH_4_ levels, beyond the effects of group and cycle day, according to our results (Table 3).

While our study design aimed to control for certain factors, we recognize that personal elements such as genetics, lifestyle and daily dietary habits could considerably affect gut microbial diversity. However, studying a specific population may also limit the generalizability of any findings. Inconsistent results across similar studies might be partially explained by these complexities, emphasizing the need for careful consideration and control of different, if not more, variables in future research. While some of our findings align with previous research, others present seemingly unique observations that could deepen our understanding of gut microbiota in the context of OCs.

## supplementary material

10.1099/jmm.0.001987 Uncited Supplementary Material 1.

## Abbreviations

ASVs: amplicon sequence variants
AUC: area under the curve
CLR: centered-log-ratio
FD: Faith's diversity
LMEMs: linear mixed effects models
NMDS: non-metric multidimensional scaling
OCs: oral contraceptives
OF: observed features
PCOS: polycystic ovary syndrome
PE: Pielou’s evenness
PERMANOVA: permutational multivariate analysis of variance
SE: Shannon entropy
T2D: type 2 diabetes
