# Supplementary material for: Influence of menstrual cycle and oral contraception on taxonomic composition and gas production in the gut microbiome
Source: J Med Microbiol. 2025 Mar 28;74(3):001987. doi: 10.1099/jmm.0.001987 (PMC11952661; doi:10.1099/jmm.0.001987)
Supplement: Uncited Supplementary Material 1. [file jmm-74-01987-s001.pdf]

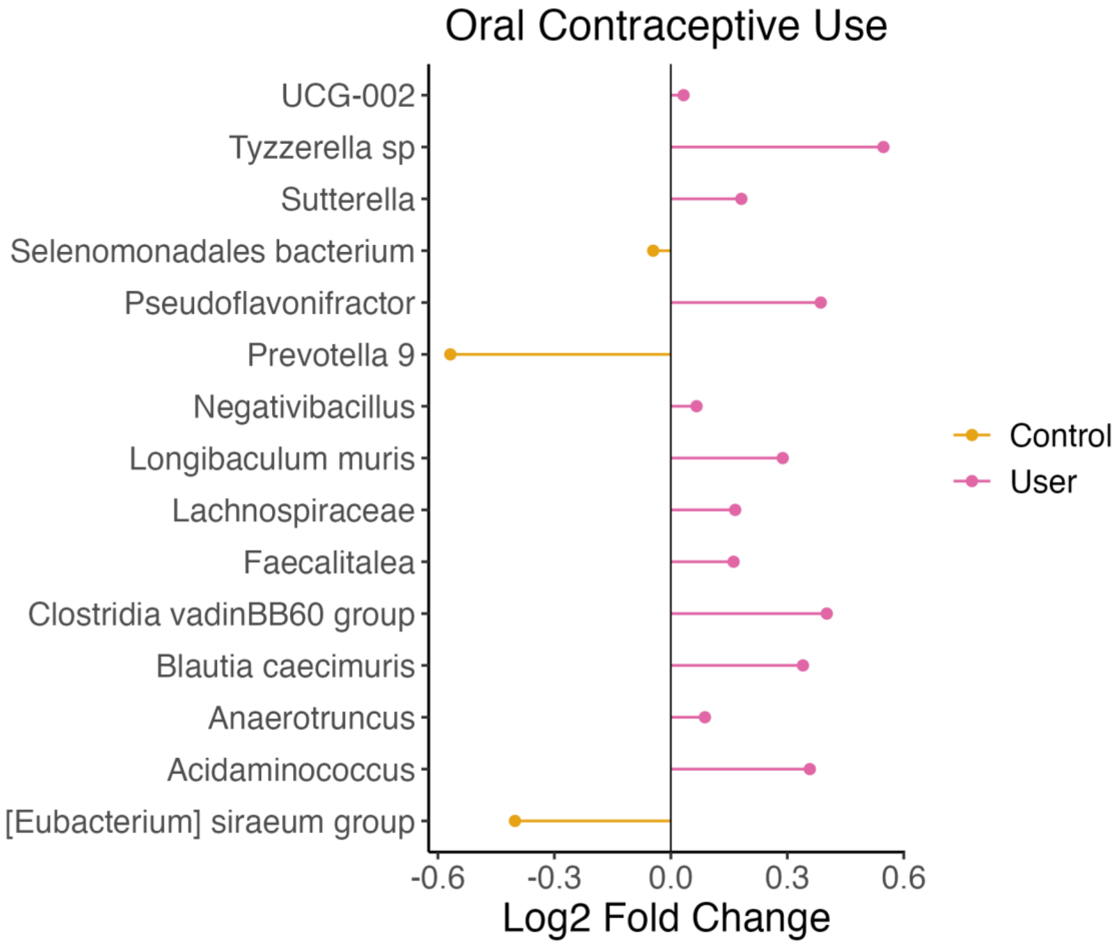

Supplementary Figure 1. Differential abundance of microbial taxa in OC user samples and controls, analyzed using ANCOMBC. Log2 fold change (LFC) values are shown for taxa identified as statistically significant (adjusted $p < 0.05$). Positive LFC values indicate higher relative abundance in the 'User' group, while negative LFC values indicate lower relative abundance when compared to the reference 'Control' group.

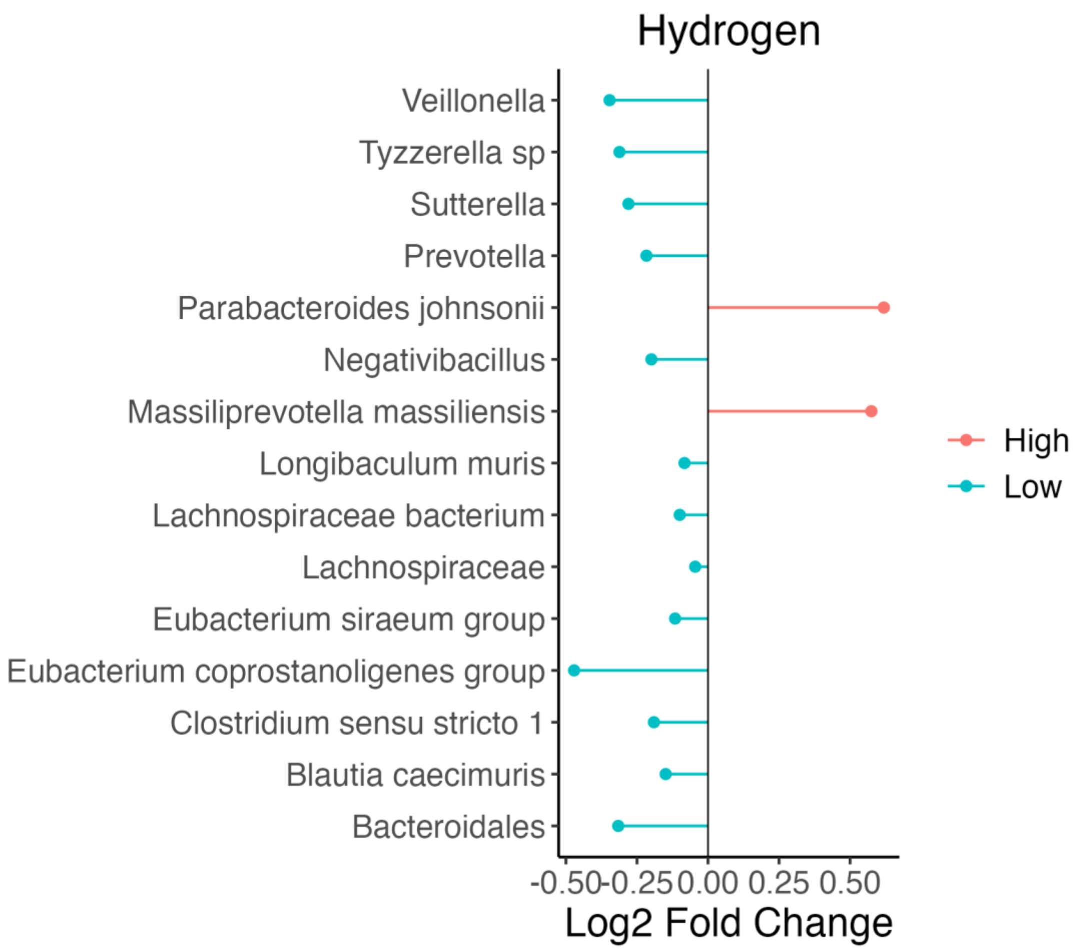

Supplementary Figure 2. Differential abundance of microbial taxa associated with high and low hydrogen levels, analyzed using ANCOMBC. Log2 fold change (LFC) values are shown for taxa identified as statistically significant (adjusted $p < 0.05$). Positive LFC values indicate higher relative abundance in the 'High' group, while negative LFC values indicate lower relative abundance when compared to the reference 'Low' group.

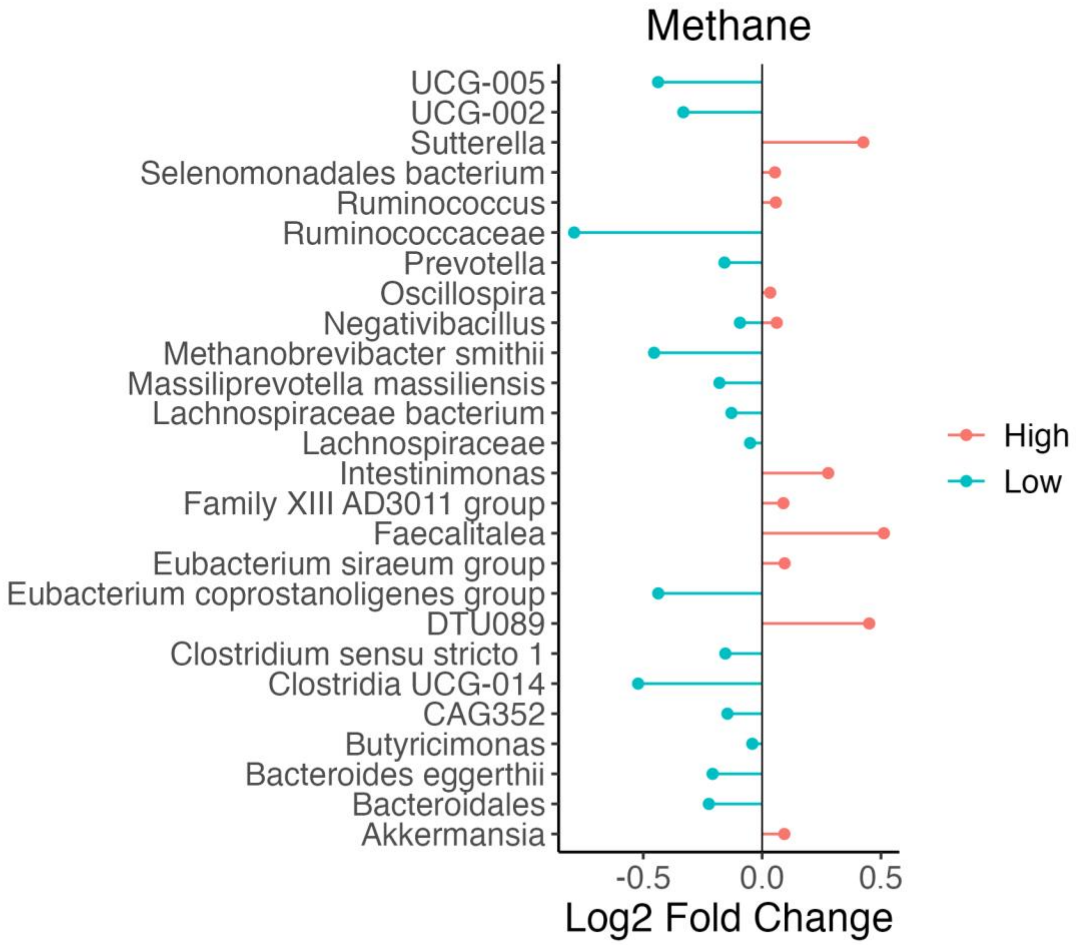

Supplementary Figure 3. Differential abundance of microbial taxa associated with high and low methane levels, analyzed using ANCOMBC. Log2 fold change (LFC) values are shown for taxa identified as statistically significant (adjusted $p < 0.05$). Positive LFC values indicate higher relative abundance in the 'High' group, while negative LFC values indicate lower relative abundance when compared to the reference 'Low' group.

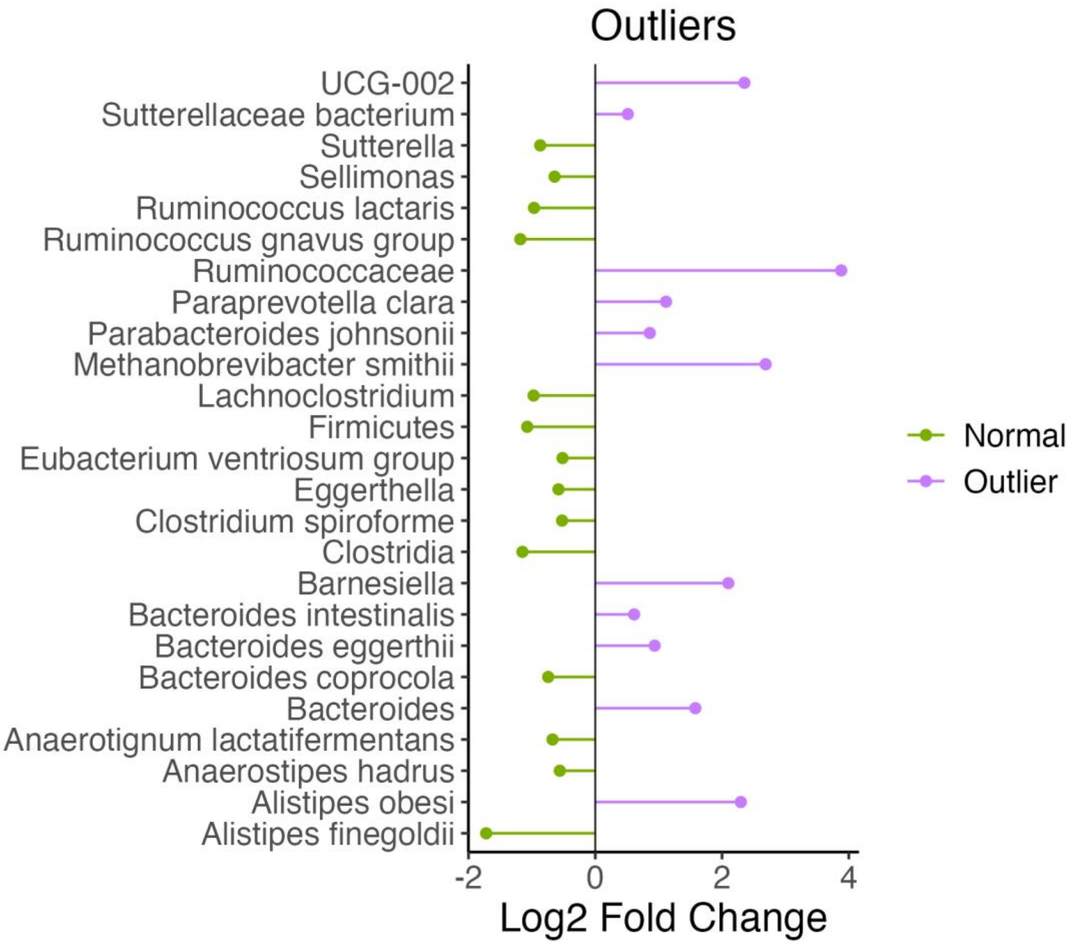

Supplementary Figure 4. Differential abundance of microbial taxa associated with outlier and normal groups, analyzed using ANCOMBC. Log2 fold change (LFC) values of ≥ 0.5 or ≤ -0.5 are shown for taxa identified as statistically significant (adjusted $p < 0.05$). Positive LFC values indicate higher relative abundance in the 'Outlier' group, while negative LFC values indicate lower relative abundance when compared to the reference 'Normal' group.

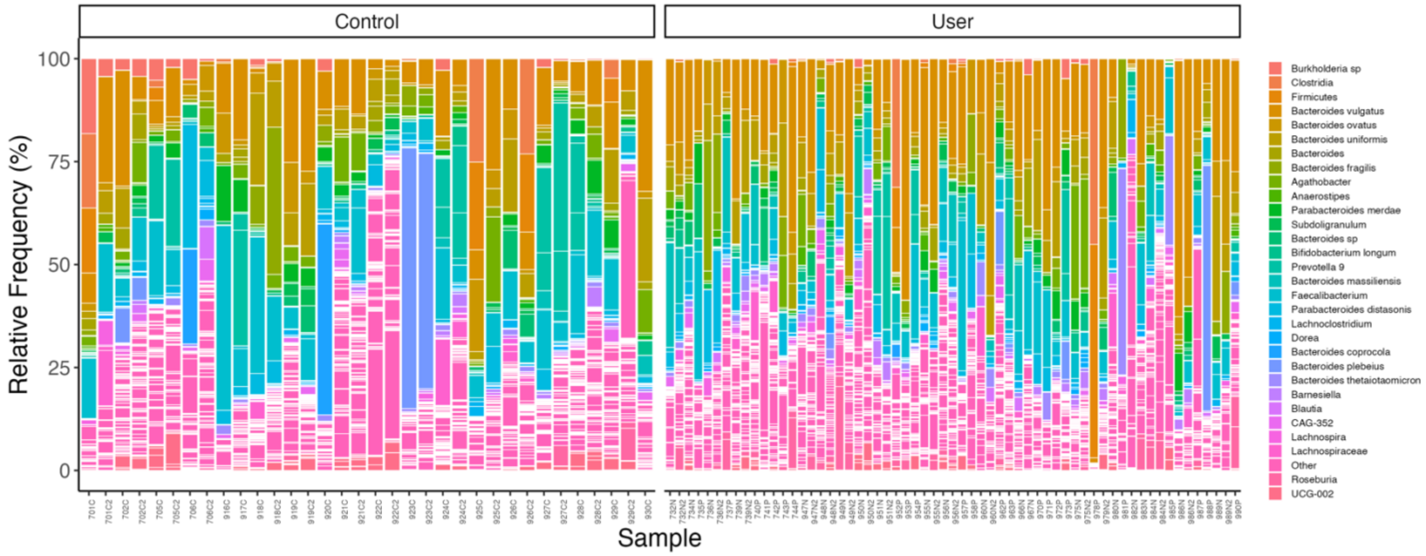

Supplementary Figure 5. Relative abundance of microbial taxa in control and OC user samples.
